# Concordance of LDL-C Estimating Equations with Direct Enzymatic Measurement in Diabetic and Prediabetic Subjects

**DOI:** 10.3390/jcm12103570

**Published:** 2023-05-20

**Authors:** Serkan Bolat, Gözde Ertürk Zararsız, Kübra Doğan, Necla Kochan, Serra I. Yerlitaş, Ahu Cephe, Gökmen Zararsız, Arrigo F. G. Cicero

**Affiliations:** 1 Department of Biochemistry, Sivas Cumhuriyet University School of Medicine, Sivas 58140, Turkey; 2 Department of Biostatistics, Erciyes University School of Medicine, Kayseri 38039, Turkey; 3 Drug Application and Research Center (ERFARMA), Erciyes University, Kayseri 38280, Turkey; 4 Department of Biochemistry, Sivas Numune Hospital, Sivas 58380, Turkey; 5 İzmir Biomedicine and Genome Center (IBG), İzmir 35340, Turkey; 6 Institutional Data Management and Analytics Unit, Erciyes University Rectorate, Kayseri 38280, Turkey; 7 Medical and Surgical Sciences Department, Alma Mater Studiorum University of Bologna, 40126 Bologna, Italy; arrigo.cicero@unibo.it; 8 Cardiovascular Medicine Unit, IRCCS AOU S. Orsola di Bologna, 40138 Bologna, Italy

**Keywords:** diabetes, prediabetes, dyslipidemia, LDL-C, LDL-C estimating equations

## Abstract

Low-density lipoprotein cholesterol (LDL-C) is a well-established biomarker in the management of dyslipidemia. Therefore, we aimed to evaluate the concordance of LDL-C-estimating equations with direct enzymatic measurement in diabetic and prediabetic populations. The data of 31,031 subjects included in the study were divided into prediabetic, diabetic, and control groups according to HbA1c values. LDL-C was measured by direct homogenous enzymatic assay and calculated by Martin–Hopkins, Martin–Hopkins extended, Friedewald, and Sampson equations. The concordance statistics between the direct measurements and estimations obtained by the equations were evaluated. All equations evaluated in the study had lower concordance with direct enzymatic measurement in diabetic and prediabetic groups compared to the non-diabetic group. Even so, the Martin–Hopkins extended approach demonstrated the highest concordance statistic in diabetic and prediabetic patients. Further, Martin–Hopkins extended was found to have the highest correlation with direct measurement compared with other equations. Over the 190 mg/dL LDL-C concentrations, the equation with the highest concordance was again Martin–Hopkins extended. In most scenarios, the Martin–Hopkins extended performed best in prediabetic and diabetic groups. Additionally, direct assay methods can be used at low values of the non-HDL-C/TG ratio (<2.4), as the performance of the equations in LDL-C estimation decreases as non-HDL-C/TG decreases.

## 1. Introduction

Diabetes mellitus (DM) is a chronic disease characterized by insulin resistance/deficiency and hyperglycemia [1]. Globally, according to the 10th edition of the IDF Diabetes Atlas the prevalence of diabetes in adults is about 10.5% [2]. Dyslipidemia, one of the significant complications of DM, is a disorder of lipoprotein metabolism in the blood, including elevated levels of total cholesterol (TC), low-density lipoprotein cholesterol (LDL-C), and triglycerides (TG), or low levels of high-density lipoprotein cholesterol (HDL-C) [3,4]. Dyslipidemia-related atherosclerotic cardiovascular disease (ASCVD) is the most common comorbidity in diabetic patients, and it is responsible for mortality in approximately 50–70% of patients [5,6,7,8,9,10,11]. Prediabetes with a high risk for type 2 diabetes (T2D) is a component of metabolic syndrome and defined as HbA1C between 5.7–6.4%. The state shares the same pathophysiological process with T2D regarding insulin resistance and/or defects in insulin secretion. Like individuals with diabetes, prediabetes has a more prominent ASCVD risk due to dyslipidemia [12]. Therefore, measuring blood lipid concentrations is crucial for objectively monitoring these patients and preventing complications.

LDL-C is an important marker, as it is the primary target in lipid-lowering therapy and the main parameter used in assessing cardiovascular disease risk and modulation of treatment for diabetic patients. Thus, LDL-C’s precise and accurate measurement is vital for preventing complications. Different methods are used to establish serum LDL-C status, including beta (β) quantification, which is used as a gold standard approach, homogenous assay techniques, and LDL-C-estimating equations. However, β quantification is not suitable for routine laboratory testing since it is time-consuming, expensive, and requires large sample volumes [13]. Therefore, many laboratories widely use either direct measurement or equations, such as Friedewald, Martin–Hopkins, and Sampson. However, because of financial reasons, the calculation techniques are broadly utilized in biochemistry laboratories instead of directly determining LDL-C.

These equations estimate LDL-C levels based on the concentrations of serum TC, TG, and HDL-C levels obtained from direct measurement. It is a well-known fact that bias and imprecision from the direct measurements and the increasing TG levels can adversely affect the accuracy of these equations [14,15,16]. Diabetic dyslipidemia is characterized by an increase in very low-density lipoprotein (VLDL) and a decrease in high-density lipoprotein (HDL) levels, resulting in an increase in triglycerides and a decrease in HDL cholesterol levels. In individuals with diabetes, there is often insulin resistance, which can contribute to the development of dyslipidemia. Insulin resistance can lead to an increase in triglycerides and non-HDL and a decrease in HDL cholesterol levels. [17,18]. Accordingly, we hypothesized that the different serum lipoprotein patterns might lead to additional erroneous effects on the accuracy of these equations. Although previous studies have evaluated the performance of the Sampson, Martin–Hopkins, and Friedewald equations in diabetic patients [19,20,21,22,23,24], none of these studies evaluated the concordance of Martin–Hopkins extended equation with direct enzymatic measurement in diabetic and prediabetic subjects. Moreover, there is no study evaluating the effect of glycemic control on the performance of Martin–Hopkins extended.

This study aimed to assess the validity of the LDL-C levels estimated by Friedewald, Martin–Hopkins, Martin–Hopkins extended, and Sampson equations with the LDL-C levels measured by direct assay in diabetic, prediabetic, and non-diabetic populations. As far as we know, no study has been conducted to evaluate these four equations in a diabetic and prediabetic populations. Therefore, our study, carried out on a large population sample, offers an exciting opportunity to advance existing knowledge regarding the use of well-known LDL-C estimation equations in diabetes and prediabetes.

## 2. Materials and Methods

### 2.1. Study Participants

The data were obtained by Sivas Cumhuriyet University Faculty of Medicine Department of Biochemistry between January 2018 and January 2021. These parameters were ordered from different polyclinics. The study was approved by the local ethics committee of Sivas Cumhuriyet University (decision number: 2022-09/03). Due to the retrospective design of the study, informed consent was not obtained from the participants. Subjects were not categorized based on fasting status. Based on HbA1c concentrations, participants (31,031) were divided into three groups as non-diabetic (<5.7% or <39 mmol/mol), prediabetic (5.7 to 6.5% or 39 to 48 mmol/mol), and diabetic (≥6.5% or >48 mmol/mol). Additionally, diabetes patients were separated into controlled (HbA1c values 6.5 to 7%) and uncontrolled (>7% or >53 mmol/mol) subgroups. In well-controlled diabetics, HbA1c levels may have values below 6.5%, in which case they may be classified as prediabetes. However, to prevent this misclassification, all past HbA1c levels of individuals with HbA1c values between 5.7% and 6.5% were also screened, and individuals with HbA1c levels ≥ 6.5% were excluded from prediabetes group. The following inclusion criteria were used: adult participants of 18 years old, both male and female participants from the diabetic group whose full lipid profile results were included. Participants who were non-diabetics, <18 years old, and with incomplete lipid profile were excluded. We also excluded subjects with iron deficiency anemia, hemoglobinopathy, thalassemia, liver disease or abnormal liver function tests, severe renal impairment or end-stage renal disease, pregnancy, and malignancy. Samples with hemolysis or icterus were rejected. Patients whose HbA1c% value is outside the values measuring range (4.2–20.1%) were excluded.

### 2.2. Biochemical Analyses

We determined HDL-C, LDL-C, TG, and TC concentrations by colorimetric enzymatic reaction using Roche Cobas 8000 c702 (Roche Diagnostics GmbH, Mannheim, Germany). LDL-C level was measured according to the following principle obtained from the method sheet of LDL-C 3rd generation kit of Roche Cobas system. Cholesterol esters and free cholesterol in LDL were measured by using the cholesterol enzymatic method. Cholesterol esterase and cholesterol oxidase in the presence of surfactants that selectively solubilize only LDL were used. The enzyme reactions to the lipoproteins other than LDL were inhibited by surfactants and a sugar compound. Cholesterol in HDL, VLDL, and chylomicron was not determined.

HbA1c was measured by turbidimetric inhibition immunoassay using the Roche Cobas c501 analyzer. The quality of analyses is ensured by performing internal quality controls, using three levels of specific internal quality control (PreciControl levels 1 and 2; Roche Diagnostics GmbH, Mannheim, Germany) and external quality control materials (Randox quality control, General Clinical Chemistry Programme, RIQAS, Antrim, UK).

Analytical CV was calculated by using two-level internal quality control results over a one-year period, and pooled CV results were established. Bias was given as the average absolute % deviation determined from the external quality assessment over the same year period. Bias and CV values were found as follows, respectively; 3.40 and 2.97 for HbA1c on Cobas c501; and 4.0 and 3.29 for TC; 3.05 and 2.90 for HDL-C; 3.63 and 3.58 for LDL-C; 3.30 and 2.86 for TG on Cobas c702.2.3.

### 2.3. Lipid Estimations

We estimated LDL-C levels using the Friedewald and recently developed equations, i.e., Martin–Hopkins, Martin–Hopkins extended, and Sampson. These equations are given below. The Friedewald equation [25] is defined as follows:

(1) $$LDL-C(F)=TC-HDL-C-\frac{TG}{5}$$

where LDL-C(F) denotes the estimated LDL-C level by Friedewald. We note that the Friedewald equation employs a fixed TG/VLDL-C ratio in estimating LDL-C levels.

The Martin–Hopkins equation [26] is defined as follows:

(2) $$LDL-C(M)=TC-HDL-C-\left(\frac{TG}{Ϛ}\right)$$

where LDL-C(M) denotes the estimated LDL-C levels by Martin–Hopkins and Ϛ is an adjustable factor used for the TG/VLDL-C ratio. In contrast to the Friedewald equation, the TG/VLDL-C ratio, which is no longer fixed, was determined using the 180 cells table described in Martin’s paper [26]. Using a similar table format to Martin–Hopkins, Martin–Hopkins extended equation (LDL-C(E)) was implemented to estimate the LDL-C levels. In the Martin–Hopkins extended equation, the data from the Turkish population were used to produce the strata-specific median TG/VLDL-C ratios in the 180 cells table (Table 1).

The Sampson equation [15], which uses the least square estimation, is defined as follows:

(3) $$LDL-C(S)=\frac{TC}{0.948}-\frac{HDL-C}{0.971}-\left(\frac{TG}{8.56}+\frac{TG\times Non-HDL-C}{2140}-\frac{{TG}^{2}}{16100}\right)-9.44$$

where LDL-C(S) denotes the estimated LDL-C levels by Sampson.

### 2.4. Statistical Analysis

In order to evaluate the concordance between LDL-C estimating equations and direct assay, the overall concordance statistic, which is calculated as the ratio of direct LDL-C (LDL-C(D)) in the same strata as estimated LDL-C based on estimated LDL-C levels (<70, 70 to 99, 100 to 129, 130 to 159, 160 to 189, and ≥190 mg/dL), was used. The overall concordance statistic was calculated separately for the non-diabetic, prediabetic, and diabetic groups. For each group, overall accuracies for LDL-C estimates were also calculated for the TG sublevels (<100, 100 to 149, 150 to 199, 200 to 399, and ≥400 mg/dL) and non-HDL-C sublevels (<100, 100 to 129, 130 to 159, 160 to 189, 190 to 219, and ≥220 mg/dL). Between-group comparisons were performed using a two-sided independent samples t-test and Mann–Whitney U test. Chi-square test was used to compare groups for sex. Within-group comparisons were performed using one-way analysis of variance (ANOVA) and Friedman tests. Bonferroni and Nemenyi tests were used simultaneously for multiple comparisons. A *p* value of <0.05 was considered statistically significant. Ordinary least squares regression analysis was used to compare the estimated and directly measured LDL-C levels, and residual error plots were created to depict the change in the difference between estimated and directly measured values. Additionally, mean absolute deviations and the coefficient of determination (R2) were computed. All statistical analyses were performed using R, version 4.0.4 (http://www.r-project.org, last access on 1 February 2023) and used packages ggplot2, ggforce, ggdist, gghalves, gridExtra, ggpubr, readxl, haven, tidyverse.

## 3. Results

### 3.1. Patient Characteristics

The study included a total of 3,1031 participants. The main characteristics of the participants are resumed in Table 2. Participants were divided into three groups according to their HbA1c levels: non-diabetic (*n* = 11,423, 36.8%), prediabetic (*n* = 9362, 30.2%), and diabetic (*n* = 10,246, 33.0%). Diabetic patients were also categorized into two categories: controlled (*n* = 2794, 27.3%) and uncontrolled (*n* = 7452, 72.7%). The diabetic group had a higher average age than the prediabetic and non-diabetic groups (*p* < 0.05). While almost half of the patients in the diabetic group were women, the rate of women in the non-diabetic group was 36.3% (*p* < 0.05). TC and non-HDL-C levels were higher in the prediabetic group compared to the other groups, whereas TG levels and TG/TC ratio were higher in the diabetic group (*p* < 0.05). The mean LDL-C for each group was 112 (90–136), 121 (96–146), and 113 (87–139) mg/dL, respectively. The mean estimated LDL-C concentrations were found to be higher in the prediabetic group than in other groups (*p* < 0.05). In contrast to LDL-C levels, the diabetic group had the highest median of remnant cholesterol measured directly.

### 3.2. Overall Concordances of the Various LDL-C Estimating Equations

**Figure 1:**
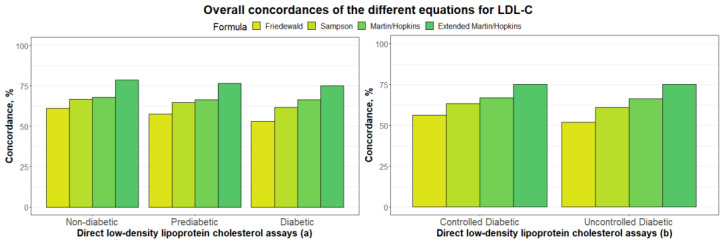
Overall concordances of the different equations for LDL-C estimation. Overall concordances of different LDL-C equations for each group (i.e., non-diabetic, prediabetic, and diabetic) are depicted in a clustered bar chart (**a**). Overall concordances of different equations for LDL-C estimation in two diabetic groups (i.e., controlled and uncontrolled) are depicted in a clustered bar chart (**b**). Each bar displays the concordance of LDL-C directly measured with LDL-C levels estimated by different formulas in different groups.

Figure 1a demonstrates the overall concordance of several LDL-C estimation algorithms for non-diabetic, prediabetic, and diabetic groups. The figure shows that the Martin–Hopkins extended method yielded the highest concordance for each group, whereas Friedewald’s equation yielded the lowest concordance. The concordance of all equations, except for the Martin–Hopkins extended equation, has significantly decreased for participants with prediabetes, and a further decrease follows this fall in the diabetic group. The overall concordance of LDL-C estimation equations for the controlled and uncontrolled diabetic categories is shown in Figure 1b. Even though the concordance of LDL-C levels was higher for individuals with controlled diabetes, the results showed no discernible change in LDL-C estimation when the Martin–Hopkins extended method was employed.

### 3.3. Overall Concordances of the Various LDL-C Estimating Equations by LDL-C Sublevels

**Figure 2:**
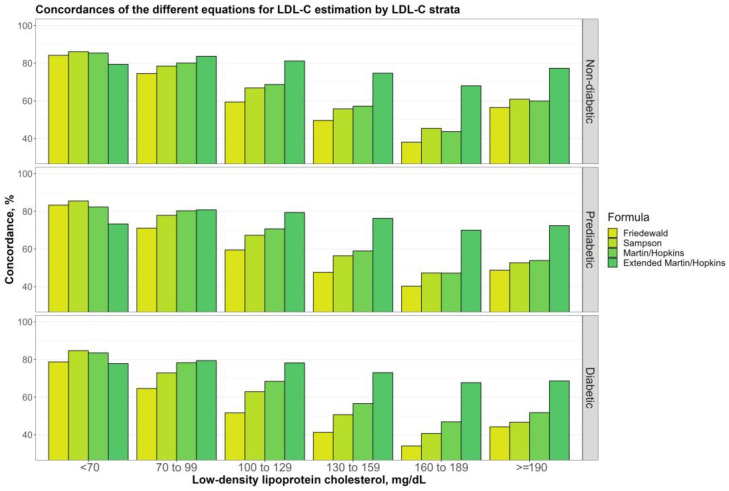
Concordances of the different equations for LDL-C estimation by LDL-C strata. Concordances of different equations for LDL-C estimation by LDL-C groups are shown in a clustered bar chart. Each bar displays the concordance of estimating LDL-C levels by direct measured LDL-C in different groups (i.e., non-diabetic, prediabetic, and diabetic).

Figure 2 demonstrates the overall concordances of the various LDL-C-estimating equations by LDL-C levels for the non-diabetic, prediabetic, and diabetic groups. The most concordant results were achieved for these groups using Sampson when LDL-C was below 70 mg/dL. Despite producing the least concordant results when LDL-C was less than 70 mg/dL, the Martin–Hopkins extended yielded the most reliable results for any participant when LDL-C was greater than 70 mg/dL. As can be seen from the figure, LDL-C levels predicted by all equations decreased until LDL-C levels reached 190 mg/dL and then increased after they did, with the diabetic group experiencing a far smaller increase. For diabetic patients, we also investigated the concordances of LDL-C estimating equations for LDL-C levels with 70 and 100 mg/dL thresholds. The Martin–Hopkins extended equation performed with the best results for LDL-C ≥ 70 mg/dL and LDL-C ≥ 100 mg/dL. In diabetic patients with lower LDL-C levels (LDL-C < 70 mg/dL and LDL-C < 100 mg/dL), the Sampson equation had the highest concordance statistics with direct assays (Table 3).

**Figure 3:**
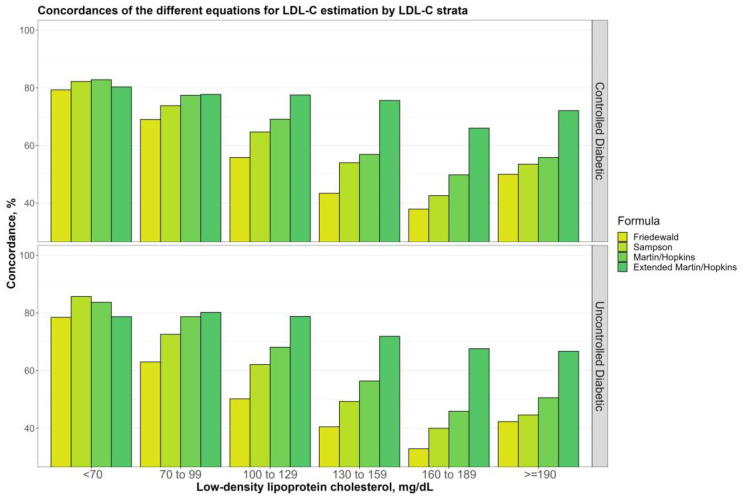
Concordances of the different equations for LDL-C estimation by LDL-C strata. Concordances of different equations for LDL-C estimation by LDL-C groups are shown in a clustered bar chart. Each bar displays the concordance of estimating LDL-C levels by direct measured LDL-C in two diabetic groups (i.e., controlled and uncontrolled).

When comparing the controlled and uncontrolled diabetic subgroup, the same trend was seen in the controlled group: LDL-C concentration was reduced until LDL-C level reached a threshold of 190 mg/dL, which began to rise (Figure 3). For the uncontrolled diabetic subgroup, though, there was no increase in the performance of LDL-C estimation using Martin–Hopkins extended when LDL-C ≥ 190 mg/dL.

### 3.4. Overall Concordances of the Various LDL-C Estimating Equations by TG Sublevels

**Figure 4:**
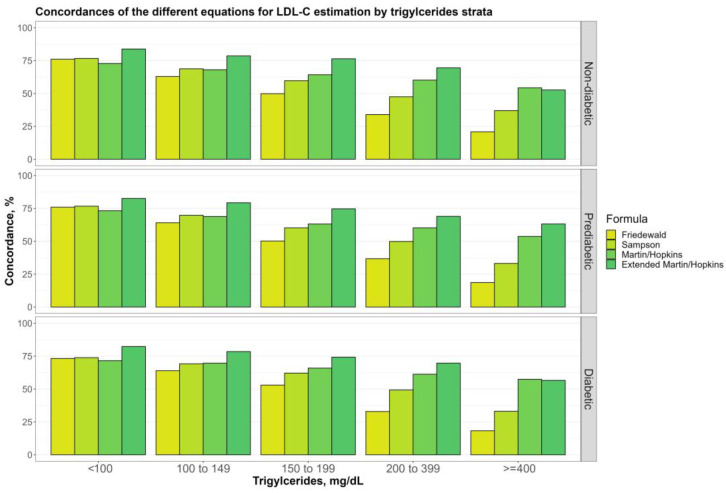
Concordance of the different equations for LDL-C estimation by triglycerides strata. Concordances of the different equations for LDL-C estimation by triglyceride groups in different groups (i.e., non-diabetic, prediabetic, and diabetic) are shown in a clustered bar chart. Each bar displays the concordance of estimating LDL-C levels by different formulas for each group of triglyceride levels by direct measured LDL-C, in different groups (i.e., non-diabetic, prediabetic, and diabetic).

**Figure 5:**
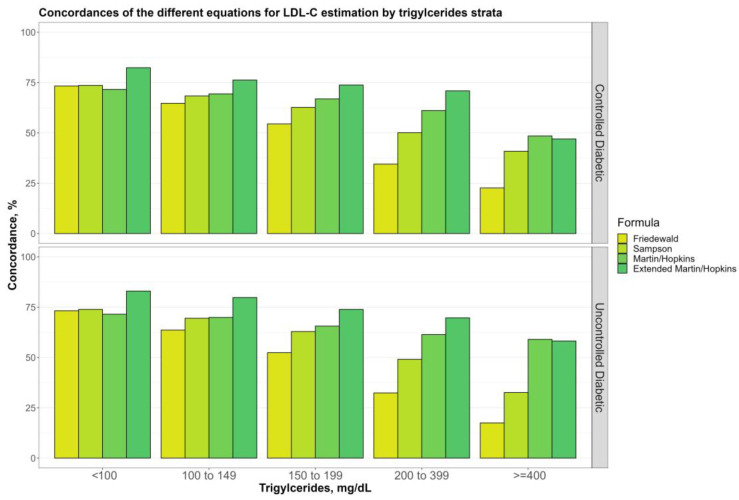
Concordance of the different equations for LDL-C estimation by triglycerides strata. Concordances of the different equations for LDL-C estimation by triglyceride groups in two diabetic groups (i.e., controlled and uncontrolled) are shown in a clustered bar chart. Each bar displays the concordance of estimating LDL-C levels by different formulas for each group of triglyceride levels by direct measured LDL-C in two diabetic groups (i.e., controlled and uncontrolled).

Figure 4 shows the overall concordances of four alternative LDL-C estimating equations by TG sublevels (100, 100 to 149, 150 to 199, 200 to 399, and ≥400 mg/dL) for non-diabetic, prediabetic, and diabetic groups. The findings demonstrated that while TG levels go up, there is a decreasing tendency for any LDL-C calculating equations. Martin–Hopkins extended performed better than the other equations for almost all TG sublevels in any group, whereas the Friedewald equation produced the least concordant results. Furthermore, Martin–Hopkins extended performed worse than Martin–Hopkins only when TG ≥ 400 mg/dL in non-diabetic and diabetic groups. Figure 5 depicts the overall concordances of LDL-C estimating equations by TG sublevels for the controlled and uncontrolled diabetic subgroups. When TG ≥ 400 mg/dL, both Martin–Hopkins and Martin–Hopkins extended equations estimated LDL-C levels more accurately for patients with uncontrolled diabetes than those with controlled diabetes. As TG levels increased, both controlled and uncontrolled diabetic subgroups showed a decreasing trend. Furthermore, Martin–Hopkins performed slightly better than Martin–Hopkins extended for any diabetic group with TG levels over 400 mg/dL.

### 3.5. Overall Concordances of the Various LDL-C Estimating Equations by Non-HDL-C Sublevels

**Figure 6:**
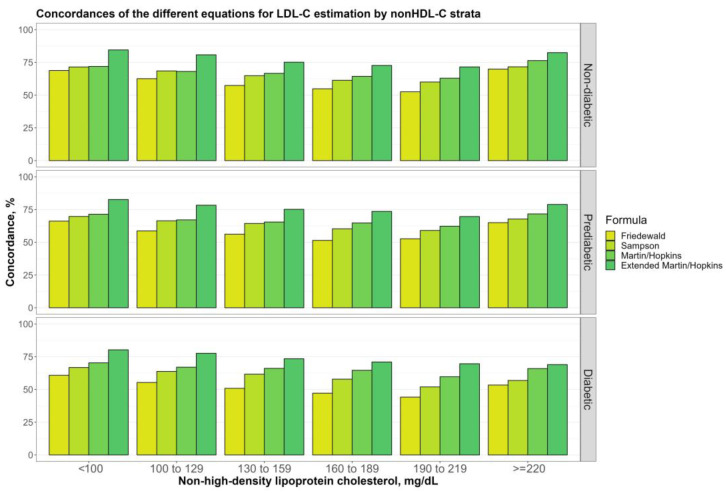
Concordances of the different equations for LDL-C estimation by non-HDL-C strata. Concordances of the different equations for LDL-C estimation by non-HDL-C groups for different groups (i.e., non-diabetic, prediabetic, and diabetic) are shown in a clustered bar chart. Each bar displays the concordance of estimating LDL-C levels by different formulas for each group of non-HDL-C levels by direct measured LDL-C in different groups (i.e., non-diabetic, prediabetic, and diabetic).

**Figure 7:**
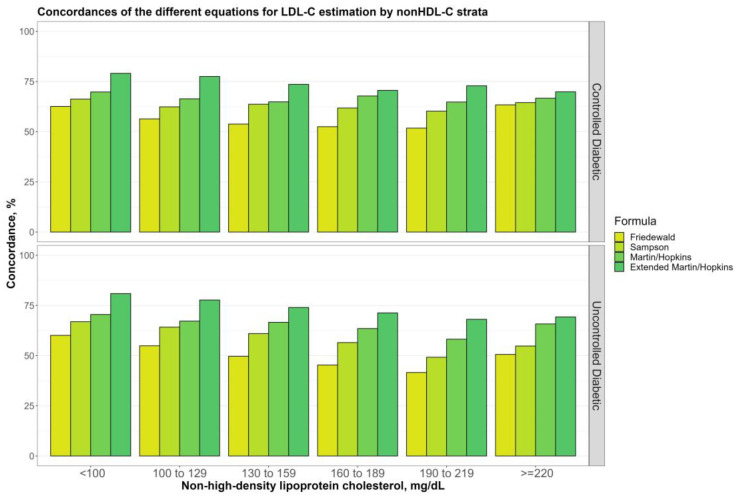
Concordances of the different equations for LDL-C estimation by non-HDL-C strata. Concordances of the different equations for LDL-C estimation by non-HDL-C groups in two diabetic groups (i.e., controlled, and uncontrolled) are shown in a clustered bar chart. Each bar displays the concordance of estimating LDL-C levels by different formulas for each group of non-HDL-C levels by direct measured LDL-C in two diabetic groups (i.e., controlled and uncontrolled).

Figure 6 shows the overall concordances of four alternative LDL-C-estimating equations by non-HDL-C sublevels (100, 100 to 129, 130 to 159, 160 to 189, 189 to 219, and ≥220 mg/dL) for non-diabetic, prediabetic, and diabetic groups. The results showed that the Martin–Hopkins extended equation performed the best for non-HDL-C sublevels in each group. It can be seen from the figure that the performance of each equation gradually decreased until the non-HDL-C level reached 220 mg/dL and then started to increase. It is clearly seen that the Friedewald equation had the lowest concordance statistics in entire groups. Furthermore, similar results and patterns were observed for each diabetic subgroup (Figure 7).

### 3.6. Overall Concordances of the Various LDL-C Estimating Equations by Non-HDL-C/TG Sublevels

**Figure 8:**
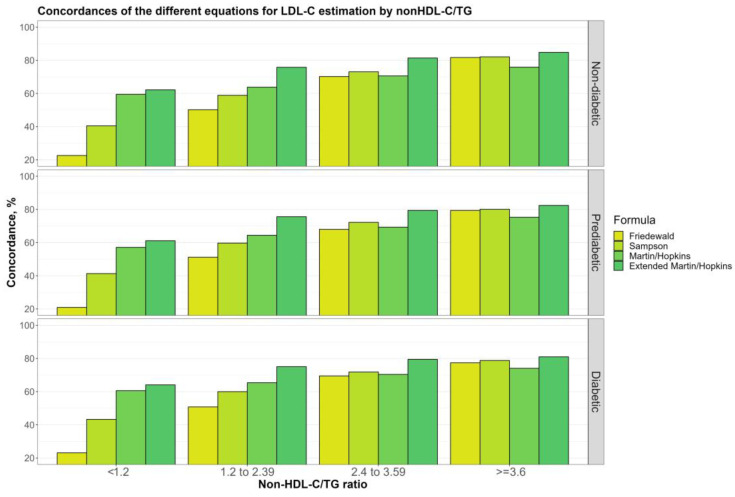
Concordances of the different equations for LDL-C estimation by non-HDL-C/TG. Concordances of different equations for LDL-C estimation by non-HDL-C/TG ratio groups are shown in a clustered bar chart. Each bar displays the concordance of estimating LDL-C levels by different formulas for each group of non-HDL-C/TG ratio levels by direct measured LDL-C in different groups (i.e., non-diabetic, prediabetic, and diabetic).

Figure 8 depicts the overall concordances of the LDL-C estimating equations by non-HDL-C/TG sublevels (<1.2, 1.2 to 2.39 mg/dL, 2.4 to 3.59, and ≥3.6 mg/dL), for non-diabetic, prediabetic, and diabetic groups. It is remarkable that the performance of all equations, especially Friedewald, decreases as the non-HDL-C/TG ratio decreases. However, this decrease was lower in Martin–Hopkins, and the Martin–Hopkins extended approaches. In individuals with non-HDL-C/TG ratios less than 1.2, the concordance statistics were around 60% for these equations.

### 3.7. Correlation between Estimated LDL-C Levels Using Different Equations and Directly Measured LDL-C Levels

**Figure 9:**
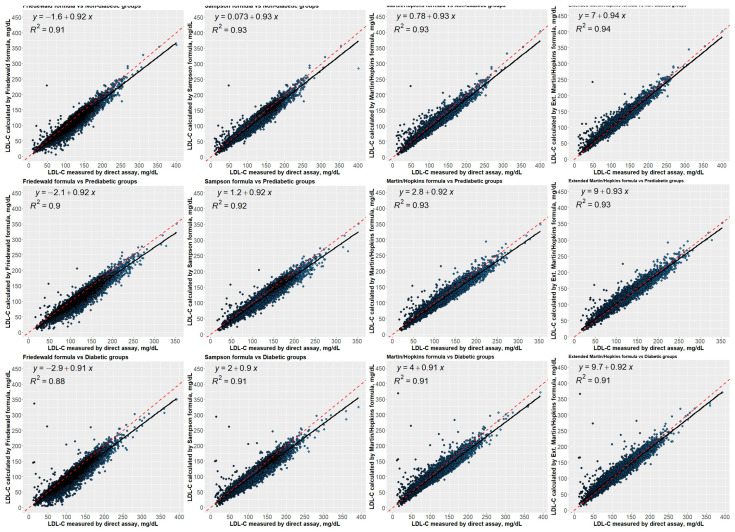
Regression analysis between LDL-C levels estimated by formulas and directly measured LDL-C levels. Correlations of LDL-C levels estimated by Friedewald, Sampson, Martin–Hopkins, and extended Martin–Hopkins formulas with LDL-C levels measured in non-diabetic, prediabetic, and diabetic groups; Red dashed line: reference curve of 45 degrees; Black line: regression curve.

**Figure 10:**
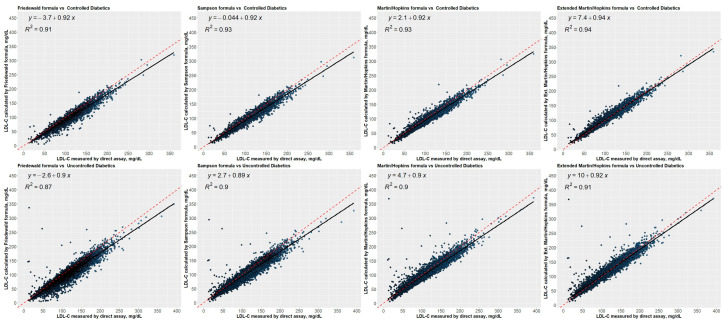
Regression analysis between LDL-C levels estimated by formulas and directly measured LDL-C levels. Correlations of LDL-C levels estimated by Friedewald, Sampson, Martin–Hopkins, and extended Martin–Hopkins formulas with LDL-C levels measured in controlled and uncontrolled diabetic groups; Red dashed line: reference curve of 45 degrees; Black line: regression curve.

Figure 9 presents the correlation between directly measured LDL-C concentrations and estimated LDL-C concentrations using different equations for each group (i.e., non-diabetic, prediabetic, and diabetic). The regression analyses revealed that all equations strongly correlated the calculated LDL-C concentrations and the directly measured LDL-C concentrations. Friedewald, on the other hand, had the weakest correlation, with R2 of 0.91, 0.90, and 0.88 for the non-diabetic, prediabetic, and diabetic groups, respectively. Compared to other equations, Martin–Hopkins extended showed a stronger correlation with the direct assay for non-diabetic patients, with an R-square of 0.94. In addition, Martin–Hopkins and Martin–Hopkins extended demonstrated similar agreement with the direct assay for prediabetic patients, with an R2 of 0.93. Moreover, for diabetic patients, all equations except Friedewald showed a similar association with the direct assay. Figure 10 presents the correlation between directly measured LDL-C concentrations and estimated LDL-C concentrations using different equations for each diabetic subgroup (i.e., controlled and uncontrolled). The Martin–Hopkins extended assay has a greater correlation with the direct assay for either diabetic group, with an R2 of 0.94 and 0.91 for the controlled and uncontrolled diabetic subgroups, respectively.

### 3.8. Residual Error Plots for LDL-C Concentrations Estimated by Different Equations concerning Direct Assay

**Figure 11:**
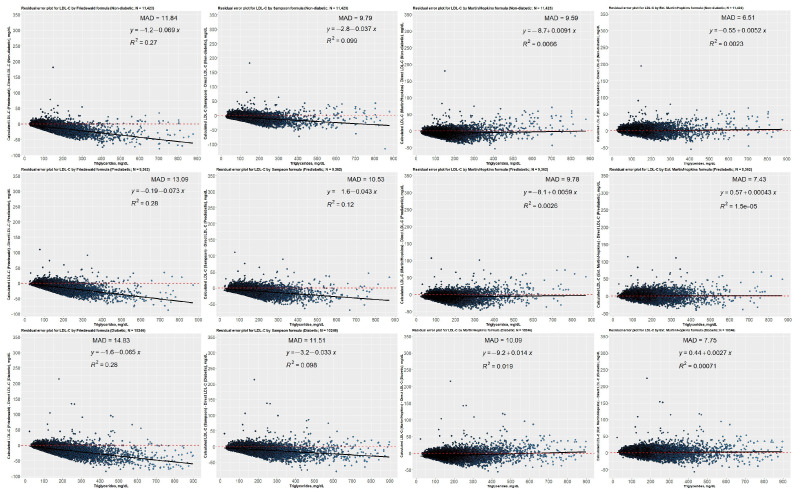
Residual error plots for LDL-C by different formulas with respect to the direct assay method. While the values on the *x*-axis represent TG levels, the values on the *y*-axis represent the difference between estimated LDL-C (as determined by Friedewald, Sampson, Martin–Hopkins, or extended Martin–Hopkins) and direct LDL-C levels (in groups non-diabetic, prediabetic, and diabetic). The mean absolute deviation (MAD) for each possible case is also provided in each panel for each dataset; Red dashed line: reference curve; Black line: regression curve.

**Figure 12:**
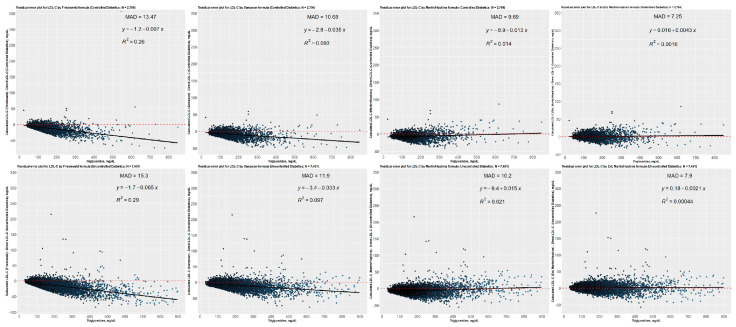
Residual error plots for LDL-C by different formulas with respect to the direct assay method. While the values on the *x*-axis represent TG levels, the values on the *y*-axis represent the difference between estimated LDL-C (as determined by Friedewald, Sampson, Martin–Hopkins, or extended Martin–Hopkins) and direct LDL-C levels (in controlled and uncontrolled diabetic groups). The mean absolute deviation (MAD) for each possible case is also provided in each panel for each dataset; Red dashed line: reference curve; Black line: regression curve.

Residual error plots were created for the non-diabetic, prediabetic, and diabetic groups to illustrate how the difference between the directly measured and estimated LDL-C concentrations varies by TG levels (Figure 11). The residual error plots for each group revealed that Friedewald and Sampson equations underestimated LDL-C levels when TG levels escalated. Even though the difference between the directly measured and predicted LDL-C values was nearly equal to zero when Martin–Hopkins equations were employed, the lowest mean absolute deviation score was achieved using the Martin–Hopkins extended equation for non-diabetic, prediabetic, or diabetic patients. Residual error plots were also created for diabetic subgroups, i.e., controlled and uncontrolled diabetic subgroups (Figure 12). When diabetic subgroups were analyzed, a very similar trend was observed for each diabetic category, namely that the Friedewald and Sampson equations underestimated LDL-C levels as TG levels increased. In contrast, the difference was close to zero for Martin–Hopkins equations. The Martin–Hopkins extended equation again recorded the lowest mean absolute deviation score for any patients with diabetes.

## 4. Discussion

Friedewald and Martin–Hopkins equations are widely used to estimate LDL-C levels. It should be noted, however, that these equations are limited by the requirement for triglyceride levels below 400 mg/dL [27]. A further limitation of the Friedewald method is the dependence on fasting serum and inaccurate determination of LDL-C levels, especially in subjects with TG ≥ 150 mg/dL or LDL-C < 70 mg/dL [15,16,17,19,20,21,22,23,24,25,26]. Several novel approaches, such as Martin–Hopkins extended and Sampson equations, have been proposed to overcome these limitations. Although previous studies evaluated the performances of the Friedewald and Martin–Hopkins equations in patients with diabetes, no study had evaluated the concordance of the Martin–Hopkins extended and Sampson equations with direct enzymatic measurement in a diabetic and prediabetic population [18,20,21,26,27,28,29,30,31,32,33]. In the present study, Friedewald, Martin–Hopkins, Martin–Hopkins extended, and Sampson equations had lower concordance with direct enzymatic measurement in diabetic and prediabetic groups than in the non-diabetic group. Impaired response to insulin is one of the main molecular mechanisms of dyslipidemia in prediabetic and diabetic patients. Furthermore, an exclusive characteristic of diabetic dyslipidemia is the increase in both apo B and other particle concentrations, such as very low-density lipoprotein (VLDL) and intermediate density lipoprotein (IDL), which distinguishes it from other types of dyslipidemia [3,4]. Accordingly, we believe that the impaired insulin response and diabetes- and prediabetes-specific serum lipoprotein patterns may further contribute to erroneous results for all these equations. It is, therefore, essential to interpret the results of these equations with caution in diabetic and prediabetic individuals.

The Martin–Hopkins extended formula takes into account non-HDL cholesterol, which includes all atherogenic lipoprotein particles in the blood, not just LDL-C, and this is particularly important in patients with high triglyceride levels, where the levels of other atherogenic lipoproteins such as VLDL-C and IDL-C are also elevated. Additionally, the formula uses a strata-specific median ratio of triglycerides to very-low-density lipoprotein cholesterol (TGs:VLDL-C) to estimate LDL-C levels in patients with triglyceride levels between 400 and 799 mg/dL. This accounts for the fact that the TGs:VLDL-C ratio varies depending on the patient’s triglyceride level, and may be more accurate than using a fixed ratio as used in other equations. Sajja et al. recently conducted a study comparing the accuracy of various methods for estimating low-density lipoprotein cholesterol (LDL-C) levels in patients with high triglyceride levels, with direct measurement of LDL-C levels [27]. The study found that the Martin–Hopkins extended equation demonstrated the strongest correlation with direct measurement, especially when using a newer TG-non-HDL-C correlation table. Similarly, we used an extended Martin–Hopkins formula by using strata-specific median ratios obtained from Turkish population data. Our study also supported this finding by demonstrating that the extended Martin–Hopkins formula exhibited the highest correlation coefficient value when compared to other equations, indicating a stronger correlation with direct enzymatic measurement of LDL-C levels.

This study detected the lowest overall concordance between direct enzymatic measurement and the Friedewald equation in diabetic and prediabetic groups. Moreover, the lowest correlation coefficient value was determined between the Friedewald equation and direct measurement compared to other equations. Although previous studies reported that the calculation of LDL-C by the Friedewald equation was not suitable in patients with DM [23,32,33,34], discordant results have been reported regarding the performance of the Friedewald equation in the diabetic population [20,22,35]. Chaen et al. found a strong correlation between the Friedewald equation and direct LDL-C measurement [20]. Razi et al. [22] and Whiting et al. [35] indicated that the Friedewald equation could be a suitable alternative for LDL-C measurement. Discordances between studies might be related to differences in the LDL-C measurement method, population number, and patient characteristics. However, considering our study’s results, we think the Friedewald equation is a less convenient method for diagnosing and managing dyslipidemia in the diabetic and prediabetic groups.

The accurate estimation of LDL-C is crucial for assessing the risk of cardiovascular disease and determining the appropriate treatment strategy for dyslipidemia in diabetes patients [5]. Following our findings, previous studies found that the Martin–Hopkins [18,22,30] and Sampson equations [24] were superior to the Friedewald equation in diabetic patients. However, the current study determined that the Martin–Hopkins extended approach has the highest overall concordance statistic in diabetic and prediabetic patients. Diabetic patients have a high risk of atherosclerotic coronary artery disease or stroke [11]. There are extremely limited studies examining the relationship between LDL-C estimating equations and cardiovascular risk factors. Overall, studies in this area have the potential to shed light on the complex interplay between diabetes, LDL-C-estimating equations, and cardiovascular health. Examining the relationship between LDL-C-estimating equations and cardiovascular risk factors may help us learn more about potential interventions that may reduce this risk.

HbA1c is the single biomarker to evaluate glycemic control, and levels above 7% are related to chronic hyperglycemic complications [36]. We confirmed that there was no effect of the glycemic control status on the concordance between extended Martin–Hopkins and direct measurement as previously reported [28]. When the equation’s performance was evaluated according to LDL-C cut points, Sampson showed higher concordances with the direct enzymatic method at the <70 and <100 mg/dL LDL-C thresholds used for the treatment target based on current guidelines [37]. However, extended Martin–Hopkins had higher concordance with the direct enzymatic method at the ≥190 mg/dL threshold, defining the high cardiovascular risk. Thus, we suggest using equations according to LDL-C cut points, treatment goals, the state of glycemic control, and cardiovascular risk categories to obtain more accurate LDL-C results and cardiac disease risk in diabetic and prediabetic subjects.

Our study found that increased TG and Non-HDL-C levels caused decreased concordances between direct enzymatic measurement and Martin–Hopkins, Martin–Hopkins extended, Friedewald, and Sampson in diabetic and prediabetic groups. Interestingly, we determined that the concordance of equations with direct enzymatic measurement was increased at ≥220 mg/dL levels of non-HDL-C in the prediabetes and non-diabetes groups. However, this pattern was not seen in the diabetes group. Earlier studies suggest that the overproduction of VLDL and IDL particles, also known as remnant cholesterol, is a fundamental component of diabetic dyslipidemia [38,39,40]. In this study, we determined higher remnant cholesterol levels in diabetic subjects than in non-diabetic and prediabetic subjects. Moreover, there are no differences between prediabetic and non-diabetic groups. Consequently, we hypothesize that the difference between these groups may be explained by remnant cholesterol contributing less to non-HDL-C levels at >220 mg/dL for prediabetic and non-diabetic individuals compared to diabetic individuals.

As in a previous study [41], we showed that decreasing non-HDL-C/TG levels caused decreased concordance between all equations and direct enzymatic methods in all groups. However, the least affected equation from decreased non-HDL-C/TG levels was Martin–Hopkins extended. Thus, we think that the accuracy of the equations may be improved by considering TG/non-HDL-C ratios. Furthermore, we determined that subjects with non-HDL-C/TG < 2.4 had decreased concordance between the Martin–Hopkins extended equation and the direct enzymatic method. This finding suggests that the non-HDL-C/TG ratio may be a novel marker in evaluating the accuracy of the Martin–Hopkins extended equation in diabetic and prediabetic populations.

As for other similar studies [42,43], the main limitation of our study is that it is based on a cross-sectional observation of a single population sample, and therefore our results should be confirmed in other ethnicities, as well. Other limitations of this study are the absence of data related the treatment of diabetes, dyslipidemia, and other metabolic diseases that could have somewhat influenced the LDL-C dosage. However, the aim of our study was to evaluate the reliability of a new LDL-C estimating formula in clinical practice, where patients usually take any kind of drugs.

## 5. Conclusions

As in the non-diabetic population, the concordance of Martin–Hopkins, Martin–Hopkins extended, Friedewald, and Sampson equations with direct enzymatic methods was affected by different conditions, including the levels of TG, LDL-C, non-HDL-C, non-HDL-C/TG, and glycemic control. The extended Martin–Hopkins equation was the best regarding overall concordance with enzymatic measurements and avoiding the effects of glycemic control. Since the decreased performance of the extended Martin–Hopkins in diabetic and prediabetic subjects with non-HDL-C/TG ratio < 2.4, we recommend using direct LDL-C measurements for these patients. Lastly, the effectiveness of the equations varies according to the level of LDL-C. Due to this, we believe that selecting an equation based on the LDL-C threshold is beneficial for obtaining more accurate results. Finally, it would be valuable to explore the long- and short-term effects of treatment on LDL-C estimation in diabetic population.

## Figures and Tables

**Table 1:** Median statistics for the ratio of triglycerides to very low-density lipoprotein cholesterol by the cross table of non-high-density lipoprotein cholesterol and triglycerides calculated from the Turkish population.

TG Levels (mg/dL)	Non-HDL-C (mg/dL)					
	<100	100–129	130–159	160–189	190–219	≥220
7–49	8.17	6.62	5.00	4.36	-	14.67
50–56	9.00	7.00	5.58	3.50	-	3.53
57–61	8.50	8.14	6.33	5.45	6.56	-
62–66	8.13	7.33	7.33	5.13	4.14	-
67–71	9.57	7.67	6.18	5.65	4.38	17.00
72–75	9.25	8.00	7.40	7.40	7.90	18.25
76–69	9.50	7.80	5.57	7.18	12.61	8.53
80–83	9.22	8.20	7.55	8.15	4.78	4.21
84–87	8.70	8.70	7.08	5.73	4.63	8.07
88–92	8.80	8.80	7.08	5.75	5.80	10.49
93–96	8.73	8.64	8.00	6.00	5.00	5.00
97–100	9.00	8.82	8.25	6.39	5.50	28.58
101–105	8.58	8.58	8.67	6.80	6.59	20.20
106–110	9.00	8.38	8.46	8.04	5.43	4.98
111–115	8.85	8.77	8.62	7.60	6.53	5.81
116–120	9.45	8.54	8.54	7.44	6.16	5.02
121–126	9.38	9.00	8.23	7.41	7.24	6.78
127–132	8.60	9.21	8.19	7.17	6.07	5.74
133–138	8.50	9.20	8.53	8.38	6.14	5.52
139–146	8.75	9.00	8.59	8.24	6.64	6.67
147–154	9.34	9.06	8.65	8.05	6.61	8.82
155–163	9.38	9.35	8.72	8.00	7.62	7.11
164–173	9.56	9.36	8.45	7.59	7.64	6.59
174–185	8.70	9.50	8.71	7.96	6.96	6.81
186–201	9.75	9.07	8.88	8.28	7.14	7.11
202–220	8.87	9.32	8.90	8.23	7.77	6.79
221–247	8.54	9.40	8.77	8.54	8.18	7.59
248–292	8.93	9.18	8.69	8.39	7.61	7.30
293–399	8.59	9.11	8.68	8.11	7.65	7.15
≥400	9.80	9.08	8.69	7.99	7.32	6.57

TG: triglycerides; HDL-C: high-density lipoprotein cholesterol; -: Median statistics were not calculated since there were no samples in this case.

**Table 2:** Patient characteristics.

Characteristic	Group				Diabetic Group		
	Non-Diabetic (*N* = 11,423)	Prediabetic (*N* = 9362)	Diabetic (*N* = 10,246)	*p*	Uncontrolled (*N* = 7452)	Controlled (*N* = 2794)	*p*
Age (years)	43.57 ± 16.48 ^a^	56.97 ± 14.19 ^b^	61.12 ± 12.43 ^c^	**<0.001**	60.78 ± 12.45	61.99 ± 12.34	**<0.001**
Sex							
Female	7276 (63.7) ^a^	5583 (59.6) ^b^	5768 (56.3) ^c^	**<0.001**	4174 (56.0)	1594 (57.1)	0.892
Male	4147 (36.3) ^a^	3779 (40.4) ^b^	4478 (43.7) ^c^		3278 (44.0)	1200 (42.9)	
Lipid values							
TC (mg/dL)	176.00 (152.00–204.00) ^a^	186.00 (157.00–215.00) ^b^	178.00 (149.00–210.00) ^c^	**<0.001**	178.00 (149.00–210.00)	178.00 (148.00–208.00)	0.164
TG (mg/dL)	116.00 (83.00–165.00) ^a^	139.00 (100.00–193.00) ^b^	155.00 (111.00–221.00) ^c^	**<0.001**	159.00 (113.00–229.00)	146.00 (107.00–201.25)	**<0.001**
HDL-C (mg/dL)	46.00 (39.00–56.00) ^a^	44.00 (37.00–52.00) ^b^	41.00 (34.75–49.00) ^c^	**<0.001**	41.00 (34.00–49.00)	42.00 (36.00–50.00)	**<0.001**
Non-HDL-C (mg/dL)	127.00 (103.00–155.00) ^a^	140.00 (113.00–169.00) ^b^	134.00 (107.00–166.00) ^c^	**<0.001**	135.00 (107.00–167.00)	134.00 (104.00–162.25)	**0.002**
TG-TC ratio	0.66 (0.50–0.93) ^a^	0.76 (0.58–1.02) ^b^	0.89 (0.67–1.22) ^c^	**<0.001**	0.92 (0.68–1.25)	0.85 (0.64–1.12)	**<0.001**
LDL-C(D) (mg/dL)	112.00 (90.00–136.00) ^a^	121.00 (96.00–146.00) ^b^	113.00 (87.00–139.00) ^a^	**<0.001**	113.00 (88.00–140.00)	113.00 (86.00–138.00)	0.467
LDL-C(F) (mg/dL)	101.00 (79.80–124.40) ^a^	108.60 (84.60–132.60) ^b^	99.00 (75.00–124.80) ^c^	**<0.001**	98.60 (74.80–124.60)	100.20 (75.40–125.25)	0.306
LDL-C(S) (mg/dL)	103.79 (82.60–127.19) ^a^	112.24 (88.37–135.85) ^b^	103.41 (79.62–128.60) ^a^	**<0.001**	103.07 (79.72–128.49)	104.20 (79.29–129.06)	0.717
LDL-C(M) (mg/dL)	104.13 (82.92–127.55) ^a^	113.30 (90.07–137.13) ^b^	105.98 (82.08–131.14) ^c^	**<0.001**	105.85 (82.67–131.37)	106.23 (80.70–130.52)	0.385
LDL-C(E) (mg/dL)	112.00 (90.33–135.68) ^a^	121.17 (97.62–145.78) ^b^	113.95 (89.36–139.42) ^c^	**<0.001**	113.58 (89.44–139.25)	114.01 (87.93–138.58)	0.612
Non-HDL-C-TG ratio	2.46 (1.82–3.23) ^a^	2.24 (1.70–2.92) ^b^	1.91 (1.43–2.52) ^c^	**<0.001**	-	-	-
Remnant-C(D) (mg/dL)	14.00 (8.00–22.00) ^a^	17.00 (10.00–26.00) ^b^	19.00 (11.00–29.00) ^c^	**<0.001**	-	-	-

Values are expressed as *N* (%), mean ± SD or median (1st–3rd quartiles). TC: total cholesterol; TG: triglycerides; HDL-C: high-density lipoprotein cholesterol, LDL-C: low-density lipoprotein cholesterol; LDL-C(D): LDL-C measured by direct assay; LDL-C (F): LDL-C calculated by Friedewald formula; LDL-C(S): LDL-C calculated by Sampson formula; LDL-C(M): LDL-C calculated by Martin/Hopkins formula; LDL-C(E): LDL-C calculated by the extended Martin–Hopkins formula; Remnant-C (D): Remnant cholesterol calculated by direct assay. Different lowercase letters (a–c) in the same line indicate a statistically significant difference among groups. Significant *p* values are shown in bold.

**Table 3:** The concordances (%) of LDL-C estimating equations in different LDL-C thresholds in diabetic patients.

LDL-C	LDL-C Estimating Equations			
	Friedewald	Sampson	Martin–Hopkins	Extended Martin–Hopkins
LDL-C				
<70 mg/dL	78.7	84.7	83.5	77.8
≥70 mg/dL	89.9	93.5	95.7	98.7
LDL-C				
<100 mg/dL	92.5	94.4	93.6	88.3
≥100 mg/dL	76.0	83.0	86.5	95.3

LDL-C: Low-density lipoprotein cholesterols.

## Data Availability

Data will be made available on reasonable request.
